# van der Waals Nanochemical Reactors

**DOI:** 10.1021/acs.nanolett.5c06176

**Published:** 2026-01-26

**Authors:** Zhaoyi Joy Zheng, Haosen Guan, Danrui Ni, Guangming Cheng, Yanyu Jia, Ipsita Das, Yue Tang, Ayelet J. Uzan-Narovlansky, Lihan Shi, Kenji Watanabe, Takashi Taniguchi, Nan Yao, Robert J. Cava, Sanfeng Wu

**Affiliations:** † Department of Physics, 6740 Princeton University, Princeton, New Jersey 08544, United States; ‡ Department of Electrical and Computer Engineering, 6740 Princeton University, Princeton, New Jersey 08544, United States; § Department of Chemistry, 6740 Princeton University, Princeton, New Jersey 08544, United States; ∥ Princeton Materials Institute, 6740 Princeton University, Princeton, New Jersey 08544, United States; ⊥ Research Center for Electronic and Optical Materials, 52747 National Institute for Materials Science, 1-1 Namiki, Tsukuba 305-0044, Japan; # Research Center for Materials Nanoarchitectonics, 52747 National Institute for Materials Science, 1-1 Namiki, Tsukuba 305-0044, Japan

**Keywords:** nanoreactors, van der Waals materials, synthesis

## Abstract

Synthesizing single crystals suitable for quantum electronic discoveries remains challenging for many emerging materials. We introduce van der Waals (vdW) stacks as nanochemical reactors for single-crystal synthesis and demonstrate their broad applicability in growing both elemental and compound crystals at the micrometer scale. By encapsulating atomically thin reactants that are stacked compactly with inert vdW layers, we achieve nanoconfined synthesis with the resulting crystals remaining encapsulated. As a proof of concept, we synthesized isolated single crystals of elemental tellurium and distinct types of Pd–Te compounds. Structural characterization confirms the high crystalline quality of the products. We observe the intrinsic semiconducting gap of tellurium and superconductivity in nonstoichiometric PdTe_1–*x*_ with a significantly reduced Te content. The concept of vdW nanoreactors is broadly generalizable, chip-integrable, well-suited to a wide range of processing conditions, and compatible with nanofabrication, offering a versatile pathway to expand the accessible landscape of quantum materials.

## Graphical abstract

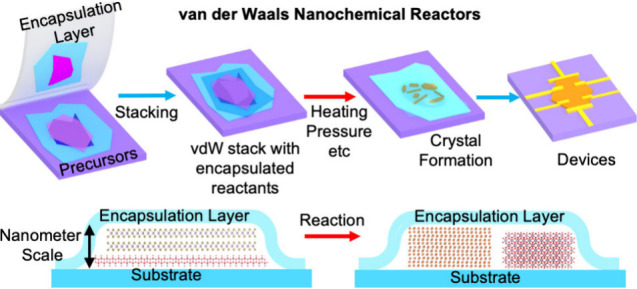

Chemical reactions in nanoconfined spaces isolated from the surroundings can be fundamentally different from those in open environments. Controlling and understanding chemical reactivity and material transformations in nano- and microscale reactors are essential to addressing challenges across multiple disciplines spanning from life science and biotechnology to catalysis and nanotechnology.^1–10^ Various nanoreactors have been explored for investigating the formation of nanoparticles and nanocrystals under confinement in different context,^9–11^ using a range of architectures including rigid nanotubes,^12–14^ polymer assemblies,^9,15^ proteins,^16,17^ mesoporous templates,^18,19^ microfluidic droplets,^20–22^ and lithographically defined patterns.^23,24^

Exploring nanoreactors for synthesizing quantum materials is, to date, a largely unexplored arena. Mature synthetic routes to inorganic crystalline materials for investigating quantum electronic phenomena are bulk-phase and thin-film growths in solid-state, solution, or vapor-based open chemical environments,^1–4^ leaving behind vast opportunities to be explored in the small scale under nanoconfinement. To advance, nanoreactors capable of accelerating quantum materials discoveries would require several key features simultaneously that are however mostly not available in previously developed nanoreactors:^9,10^ (1) The final product is in a single-crystal form with a micrometer size at least in one direction (i.e., not merely nanoparticles); (2) the reaction is generalizable to various combinations of reactants with controlled stoichiometric compositions for synthesizing a broad class of materials; (3) the nanoreactors can support harsh synthetic conditions, such as high temperatures or high pressures, often necessary for quantum materials synthesis; (4) the product crystal is isolated from unwanted substances; (5) the synthesis is compatible with further nanofabrication processes for constructing devices from the as-grown crystals, essential for investigating its quantum electronic or photonics properties. In this work, we introduce the concept of van der Waals (vdW) nanoreactors (Figure 1), which feature all of the aspects mentioned above.

**Figure 1:**
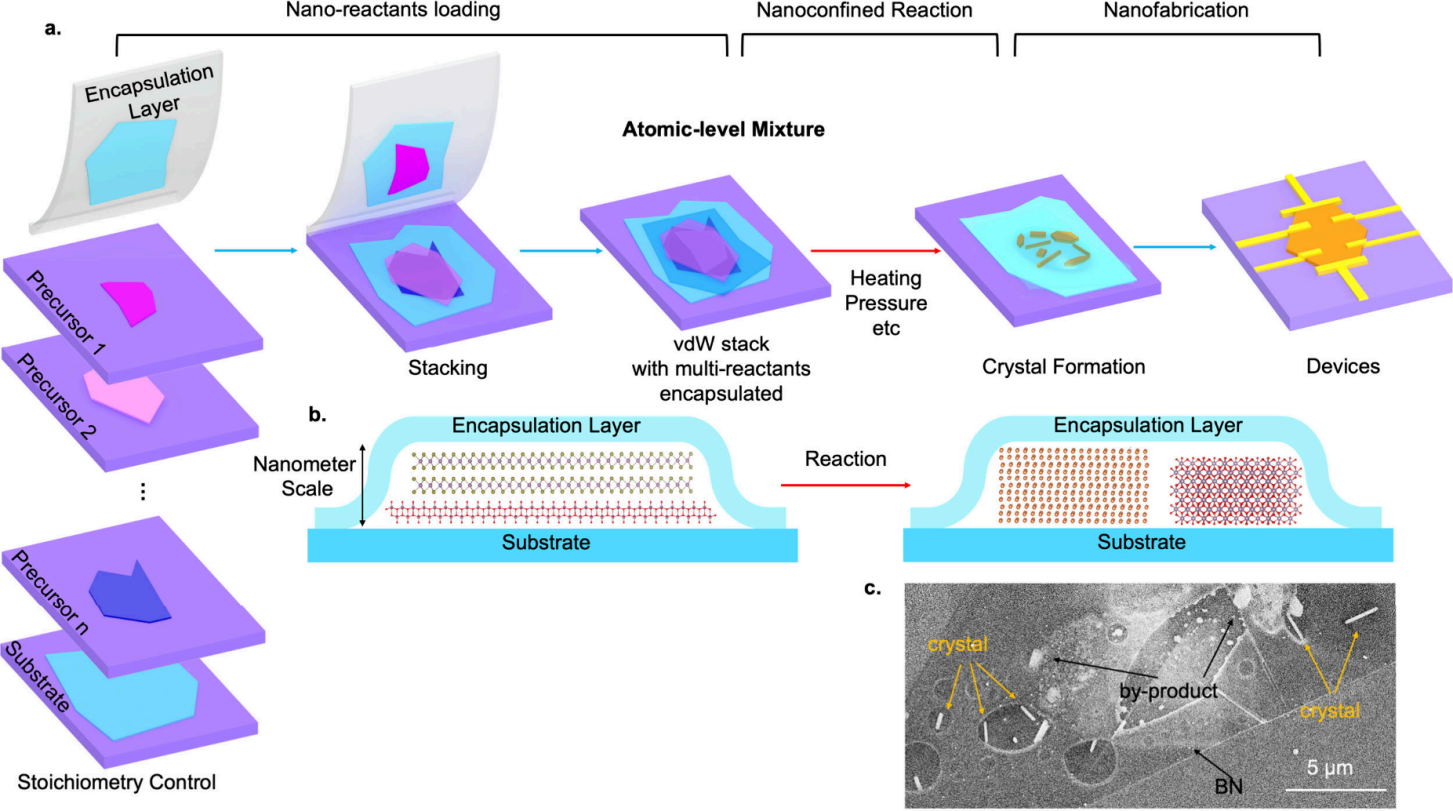
van der Waals nanoreactors. **a**, A schematic flowchart for creating vdW nanoreactors, introducing nanoconfined reactions, and fabricating nanodevices for investigating electronics properties of as-grown crystals. **b**, A cross-sectional illustration of a representative possible nanoreactor before and after the chemical reaction. The encapsulation layer is a chemically inert vdW material, such as hBN. The substrate can be hBN or other typical rigid substrates such as SiO_2_, sapphire, etc. **c**, An SEM image of a representative vdW nanoreactor after the reaction, showing the formation of single crystals and byproduct materials. This specific vdW nanoreactor is created following a procedure similar to that described in Figure 2. The crystals synthesized are high-quality tellurium nanowires, whereas the substrate is Si/SiO_2_ and the encapsulation layer is hBN.

vdW stacks of atomically thin 2D crystals have been one of the foci in condensed matter research since they enable the integration of distinct materials and physical phenomena at the atomic scale with striking modification of low-energy electronic structures through, for example, moiré quantum engineering.^5–7,25–29^ Emphasis has been so far placed on the modulation of the physical properties, instead of chemical reactivity, of the materials encapsulated in vdW stacks. Here we change the mindset to view vdW stacks as an extraordinary mixture of atomically thin reactants that are packed closely together at the atomic scale (Figure 1a), unprecedented to conventional solid-state grinding methods used in bulk growth. An inert layer, such as hexagonal boron nitride (hBN), can be used to encapsulate and isolate the mixture from the environment, forming a confined nanoreactor (Figure 1b), which allows for high-temperature treatments^30^ and prevents the loss of masses to the environment or contamination. Figure 1a illustrates the flowchart for creating vdW nanoreactors. Atomically thin layered reactants are first exfoliated onto a substrate (e.g., the typical SiO_2_/Si), which may or may not undergo further processes (such as oxidization, etc.) to modify its chemical compositions. A series of precursor layers, together with the encapsulation layers, can then be picked up subsequently and stacked to form a vdW stack using the standard transfer techniques used in the 2D material community. Chemical reactions will be triggered inside the stack by methods such as heating (used in this work) and/or pressure (Figures 1b,c). The hBN encapsulation layer can support reactions up to ∼1000 °C^30^ (Figure S1), allowing for its use in the synthesis of a wide class of materials. We note that due to nanoconfinement, reactions between the atomically thin and compact reactants within the vdW stacks can occur at temperatures significantly lower than that required for conventional crystal growth.

We highlight two important aspects associated with vdW nanoreactors. First, the choices for precursor layers are immensely diverse. It has been estimated that more than 1,000 layered crystals can be exfoliated down to the 2D limit,^31–33^ each of which may be further chemically treated to form derived structures. All of these can potentially be used as reactants. Thin film depositions can also introduce nonlayered materials, such a thin layer of metal, to be included. The ability to control atomic composition and stoichiometry in the vdW nanoreactors is hence exceptional. Second, techniques for characterizing the final crystals, including both atomic structures and their electronic and optical properties, are readily available thanks to the developments for 2D materials. For instance, crystals formed inside the vdW stacks can be readily turned into electrical transport devices by using established 2D nanofabrication methods. Below we demonstrate the methodology and the applications of vdW nanoreactors by achieving the growth and characterization of distinct representative crystals.

We begin by illustrating the creation of a vdW nanoreactor consisting of an hBN/MoTe_2_/oxidized MoTe_2_/hBN stack where the reactants are intrinsic few-layer 2H-MoTe_2_ and its oxide (Figure 2a). The oxidization of MoTe_2_ on the hBN bottom layer is achieved by thermal annealing at 280 °C in an oxygen environment, resulting in an oxygen-rich amorphous Mo–Te–O mixture that is consistent with the formation of MoO_3_ and TeO_*x*_ (Figure S2). The fully encapsulated stack is then heat-treated at 350 °C for about 30 min. An optical image of a stack (Growth T1) after reaction is shown in Figure 2b. A bubble is formed between the two hBN layers, likely caused by the vapor pressure developed due to, for example, sublimation of the oxides (such as MoO_3_) inside the vdW stack at high temperatures. Inside or near the bubble, wire-like new materials form, which we confirm to be tellurium single-crystalline wires. The process is highly reproducible, as shown in Figures 2c,d for another two separate growths (T2 and T3), all yielding similar results. Scanning electron microscope (SEM) images shown in the bottom panels of Figures 2c,d, reveal clearly the long rectangular morphology expected for the quasi-1D crystal structure of tellurium (Figures 2e,f).

**Figure 2:**
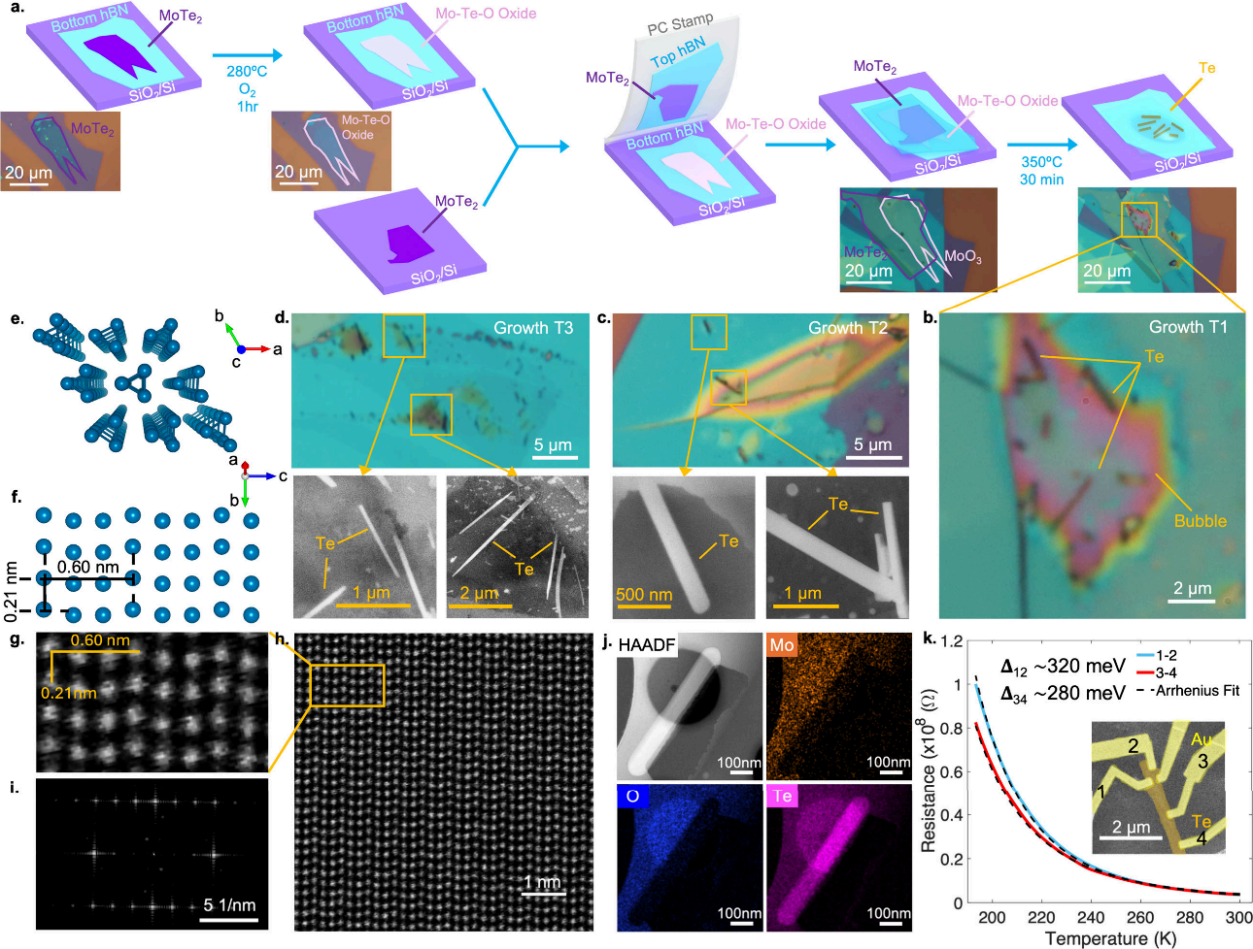
vdW nanoreactors for synthesizing tellurium single crystals. **a**, Illustration of the workflow for designing and creating the nanoreactors and the reactions. Insets are optical images of the flakes, outlined by the solid line along the boundaries, used in Growth T1 in selected steps. **b**, An optical image of the nanoreactor after the reaction. The final products are multiple separated Te wires, some of which some are indicated. The bubble is an hBN tent structure formed during the growth. **c.**, Top: The same optical image was taken for another independent growth (T2) after a similar procedure. Bottom: high-resolution SEM images taken at locations indicated in the top panel. **d**, The same as **c** but for another independent growth, T3, showing that the reaction is highly reproducible. **e** and **f**, The crystal structure of tellurium, viewed from different angles as illustrated by the compass. **g**, An atomic resolution STEM image of a typical as-grown Te wire showing matched crystal structure and interatomic distances. **h**, The same STEM image but with a larger area, showing high crystallinity. Each of the Te’s synthesized in the reactors are single crystals. **i**, FFT of the real space image showing the characteristic diffraction spots. **j**, EDX analysis of a chosen wire (with an HAADF image shown at the top left panel), showing both the clean Te wire and the formation of oxides nearby. **k**, The transport measurements of the Te wire, showing an activated behavior of the resistance measured as a function of temperature (solid lines), taken from two different pairs of contacts. The Arrhenius fit (dashed black lines) to both curves yields an activation gap, as indicated. Inset is a false-color SEM image of the device after the deposition of Au electrodes.

To confirm the high crystallinity of the product, we perform atomic-resolution scanning transmission electron (S/TEM) microscope studies on the vdW stack after reaction (see Figure S3 for the fabrication process of the TEM samples). The results are shown in Figures 2g–i, where the Te atoms in the wire are clearly revealed, confirming the crystal structure consistent with that of a tellurium single crystal (Figure 2f). No single defect appears in this observation (Figure 2i). We further perform elemental mapping using energy-dispersive X-ray spectroscopy (EDX) around a selected wire, as shown in Figure 2j, where Mo and O atoms are absent at the location of the Te crystal while forming substances nearby. The essential reaction process in this vdW nanoreactor may be approximately described as MoTe_2_ + MoO_3_ → Te + MoO_2_ + .... We emphasize that such a process occurs only because of the nanoconfinement by the hBN layer, without which all vaporized substances at high temperatures will disseminate into the open environment (Figure S1).

Elemental tellurium crystals have recently attracted substantial interest due to their potential applications in electronics,^34–38^ thermoelectricity,^39^ chiral materials,^37,38^ and nanoprostheses.^40^ Intrinsic tellurium is a semiconductor with a bandgap of ∼340 meV.^41^ However, recent experiments using solution-based growth typically yield a slight hole doping, presumably attributed to Te vacancies.^37,38^ To examine the electrical transport properties of the Te wire created in our nanoreactors, we deposited metal contacts onto the wire after etching the hBN layer only at the contact region (the noncontact region of Te is still protected by hBN), employing typical electron beam nanolithography processes. The SEM image of a device is shown as an inset in Figure 2k. We observe activated transport behavior in the resistance–temperature dependence of as-grown tellurium. The Arrhenius fits yield bandgaps of approximately 320 and 280 meV from two distinct contact regions (Figure 2k), both close to the intrinsic value. The transport data indicate substantially reduced vacancies or impurities in the sample. The observation not only confirms the semiconducting nature of our as-grown tellurium but also implies excellent quality of the crystals obtained in our nanoreactor approach.

To demonstrate the versatility of the vdW nanoreactors, we now modify the previous reaction by introducing Pd into the reaction, such that the reaction may be engineered toward producing distinct crystals, with a target of Pd–Te binary compound in mind. We introduce Pd by simply replacing MoTe_2_ with Pd_7_MoTe_2_ (Figure 3a), with a hypothetical synthetic path of Pd_7_MoTe_2_ + MoO_3_ → Pd_*x*_Te_*y*_ + MoO_2_ + .... The creation of an atomically thin layer of Pd_7_MoTe_2_ follows the procedure developed in previous works,^42–44^ which discovered that 2D metal of Pd forms upon heat treatment when a Pd source is in contact with 2D MoTe_2_ layers. The 2D Pd is precisely 7 atomic layers per MoTe_2_ layer, forming a new compound of Pd_7_MoTe_2_. To form a vdW nanoreactor, we transfer a layer of Pd_7_MoTe_2_ onto oxidized MoTe_2_ as reactant, fully encapsulated by top and bottom hBN. We then heat the stack to about 400 °C in a typical thermal annealing process (see the Methods), after which the result is examined under microscopes.

**Figure 3:**
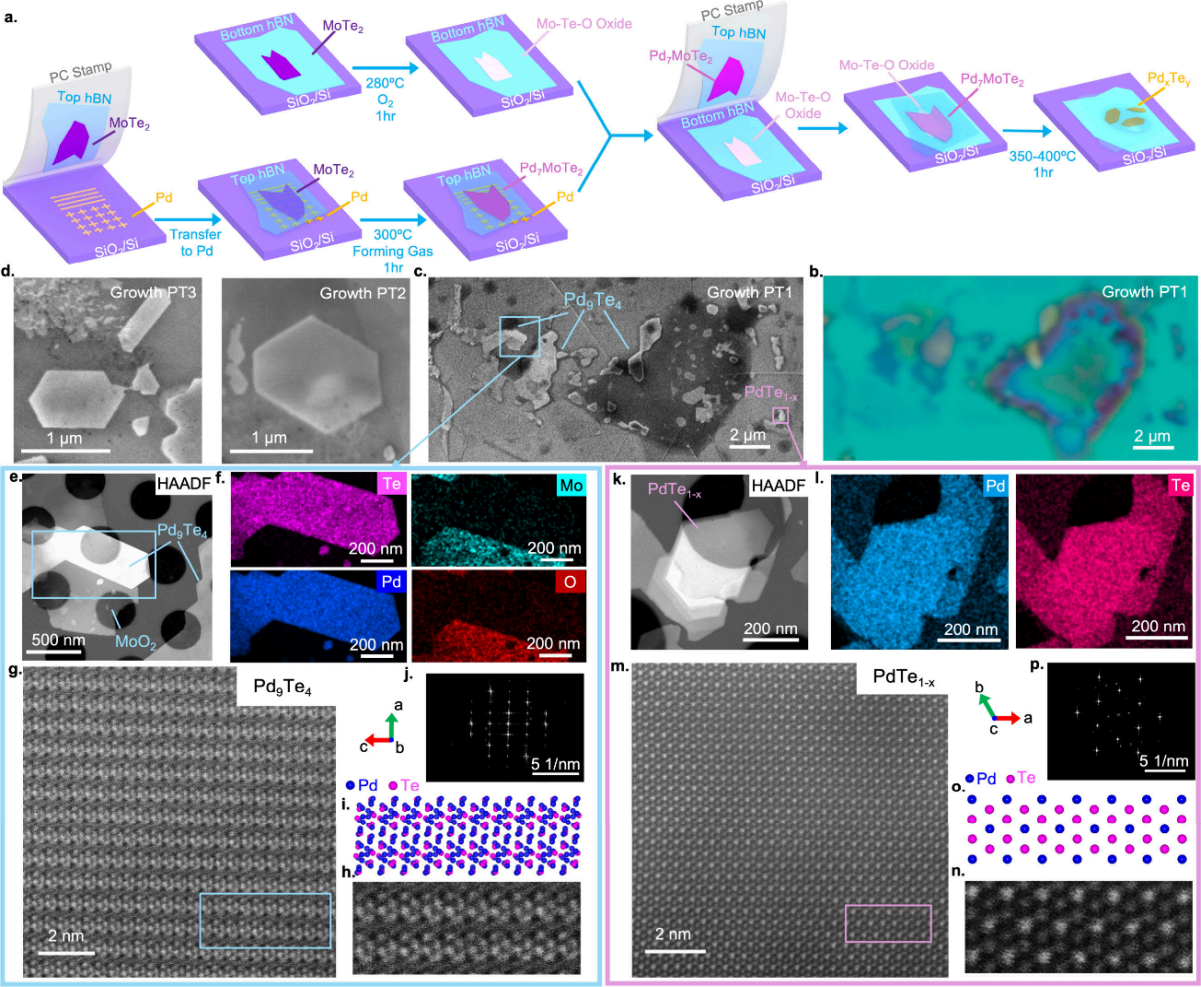
van der Waals nanoreactors for synthesizing Pd–Te crystals. **a**, Illustration of the workflow for designing and creating the corresponding nanoreactors and the reactions. **b**, Optical image of the vdW stack after the reactions (Growth PT1). **c**, A high-resolution SEM image of the same stack as **b**. **d**, SEM images of two representative crystalline flakes obtained respectively from another two independently created vdW nanoreactors and reactions, following a similar procedure. **e**, A HAADF image of a region highlighted in the blue square shown in **c**. **f**, EDX elemental mapping, with the element indicated in each panel, showing the corresponding compositions in the flakes at areas indicated by the blue rectangle line shown in **e**. The corresponding Pd–Te compounds are closely approximate to Pd_9_Te_4_, formed together with the nearby thin-film compound that is approximately MoO_2_. **g**, A representative STEM image with atomic resolution of as-grown Pd_9_Te_4_. **h**, A zoom-in plot of the same data in the blue rectangle shown in **g**, highlighting detailed features. **i**, The crystal structure of Pd_9_Te_4_, showing a pattern matched to the data in **h**. **j**, FFT of the real space image showing the characteristic spots of Pd_9_Te_4_. **k**, A HAADF image of a region highlighted in the purple square shown in **c**. **l**, EDX elemental mapping, with the element indicated in each panel, showing the compositions of the flake shown in **k**. The flake consists of Pd and Te in a ratio of about 55%:45%, indicating a nonstoichiometric PdTe_1–*x*_ with *x* ≈ 0.18. **m**, A representative STEM image with atomic resolution of as-grown PdTe_1–*x*_. **n**, A zoom-in plot of the same data in the purple rectangle shown in **m**, **o**, The modeled crystal structure of PdTe_1–*x*_, which closely resembles that of the PdTe hexagonal structure with a space group *P*6_3_/*mmc*, matching the pattern of **n**. **p**, FFT of the real space image showing the characteristic lengths of PdTe_1–*x*_.

Instead of wires, we now find crystals of very different morphologies (often elongated hexagons), as shown in Figures 3b–d for three different samples (Growth PT1–3), already implying that a different type of crystal is formed. STEM plan-view examination of location 1 in Growth PT1 (indicated by the blue square in Figure 3c) after reactions is shown in Figures 3e–j, where two products are clearly identified. The brightest area with sharp edges seen in the high-angle annular dark-field (HAADF) image (Figure 3e) corresponds to a binary crystal formed by Pd and Te, as revealed by EDX elemental mapping (Figures 3f). There is MoO_2_ formation nearby, as seen clearly in the Mo and O maps. More EDX analysis can be found in Figure S4. Atomic resolution STEM images show that the Pd–Te compound formed here develops excellent crystallinity, where the atomic structure can be identified as that of Pd_9_Te_4_ (Figures 3g–j).

It is known that the Pd–Te compounds exhibit a variety of phases with different stoichiometric compositions.^45,46^ In addition to the formation of Pd_9_Te_4_ crystals in this nanoreactor (Growth PT1), we indeed observed other binary species. As highlighted in location 2 of Growth PT1 (indicated by the purple square in Figure 3c), there is formation of another phase in this vdW nanoreactor. Its structural characterization is shown in Figures 3k–q, where EDX mapping confirms the binary composition with an atomic ratio of Pd:Te to be about 55%:45%. However, Pd_11_Te_9_ does not exist as a standalone stoichiometric phase in the Pd–Te phase diagram.^45,46^ Atomic resolution images (Figures 3m–p) reveal that the lattice structure closely resembles that of PdTe (similar to the hexagonal NiAs structure with the space group *P*6_3_/*mmc*). We hence attribute this phase as PdTe_1–*x*_ with a substantially reduced content of Te (*x* ≈ 0.18). We are not aware of other reports that produce this much reduction of Te. We note that the lattice of PdTe could retain its form likely thanks to the relatively low growth temperature used in our reaction and that the Pd richness is reasonable in our nanoreactors because Pd is significantly more abundant in the reactants loaded initially. In the next section, we reproduce the growth of this PdTe_0.82_ compound and perform careful transport characterization.

In Growth PT4, we reproduced the growth of nonstoichiometric PdTe_1–*x*_ (*x* ≈ 0.18) that is large enough for creating transport devices as seen in Figure 4a, which shows a false-colored SEM image of the device after depositing gold electrodes. We first present data confirming the high-quality crystal structure using cross-section STEM performed on the same device after transport measurement (Figures 4b–d). Followed by a cut of the device along the black dashed line shown in Figure 4a using a focused ion beam, we verify again that the atomic composition is ∼55%:45% (Figure 4b). Atomic resolution STEM images confirm that the entire compound is a uniform single crystal, with an indistinguishable lattice at different locations (Figures 4d and Figure S5). This lattice is again consistent with that of PdTe viewed from the cross-sectional angle shown in Figure 4c. We hence confirm that the same PdTe_1–*x*_ with *x* ≈ 0.18 is selected.

**Figure 4:**
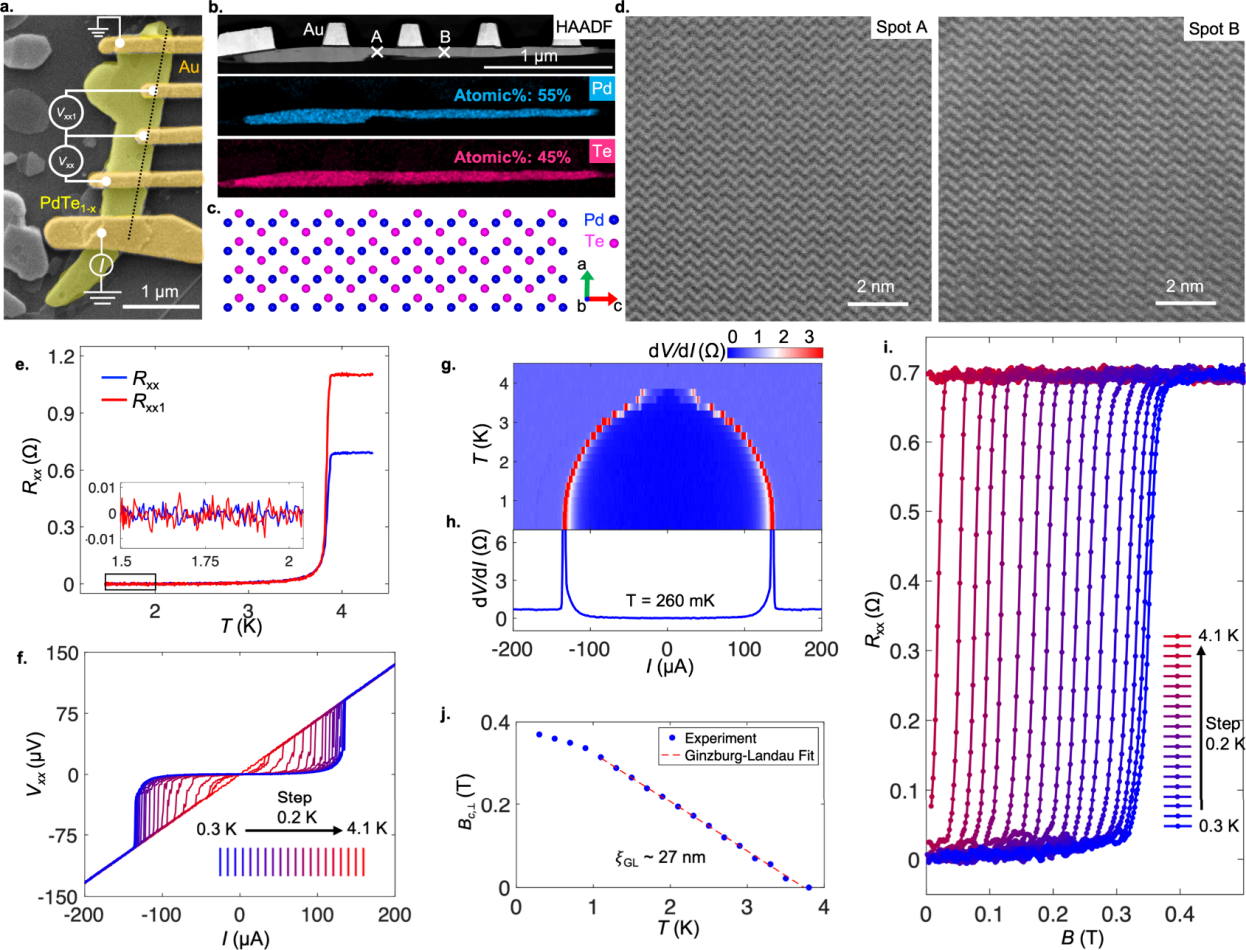
Superconductivity in PdTe_1–*x*_ (*x* ≈ 0.18). **a**, A false-colored SEM image of a device fabricated from as-grown PdTe_1–*x*_ with gold (Au) electrodes deposited for electrical measurements. **b**, Cross-section EDX analysis of the device, followed by a FIB cut along the dashed line in the same device after the transport measurement. Top: HAADF image of the cross section, showing both the Au electrodes and PdTe_1–*x*_ crystal. Middle: EDX elemental mapping of Pd. Bottom: elemental mapping of Te. The observed atomic ratio is indicated, which is consistent with the top view of EDX mapping shown in Figure 3l. **c**, The modeled crystal structure (following PdTe) viewed from a different angle indicated by the compass. **d**, Two representative STEM images with atomic resolution taken respectively at two distinct locations indicated in **b** (spots A and B), showing identical structures that are consistent with **c**. These data suggest that PdTe_1–*x*_ exhibits a highly uniform crystal structure. **e**, Resistance of the crystal as a function of temperature. The measurement configuration for all transport data is shown in **a**. The two curves correspond to data taken from the two different contact pairs, respectively. Inset shows the zero-resistance value measured at a low *T*. **f,** *IV* characteristic curves taken at various *T* values indicated. **g,** Differential resistance d*V*/d*I* as a function of applied DC current (*I*), taken at different *T*, showing the *T*-dependent critical currents. **h**, d*V*/d*I* vs *I* taken at *T* = 260 mK. **i,** *R*_*xx*_ as a function of *B*_⊥_, taken at various *T* as indicated. **j**, Extracted critical magnetic field *B*_c,⊥_ as a function of *T*. The dashed red line is the linear GL fit to the data.

We present the quantum transport properties of this PdTe_0.82_ crystal with a measurement configuration shown in Figure 4a. The crystal displays metallic behavior with a four-probe resistance *R*_*xx*_ on the order of 1 Ω at room temperature. Upon cooling, the resistance slowly decreases and suddenly drops to zero below a critical temperature, *T*_c_, of ∼3.8 K (Figure 4e), signifying the formation of superconductivity. Figure 4f plots the *IV* curves taken at different temperature (*T*) below and above the transition, revealing the typical nonlinear characteristics expected for a superconductor. Differential resistance d*V*/d*I* (Figures 4g,h) highlight the critical current of ∼130 uA at low *T*, a value that decreases upon warming up the sample. The application of external magnetic fields, *B*_⊥_, perpendicular to the plane, suppresses the superconductivity (Figure 4i) above a critical field of ∼380 mT at low *T*. We extract the critical magnetic field, *B*_c,⊥_, as a function of temperature (Figure 4j), which follows the linear Ginzburg–Landau (GL) form *B*_c,⊥_ = Φ_0_/(2π $\xi_{\mathrm{GL}}^{2}$ )­(1 – *T*/*T*_c_) near *T*_c_, where ξ_GL_ is the extrapolated GL coherence length at zero temperature and Φ_0_ is the flux quantum. We found ξ_GL_ to be approximately 27 nm. Superconductivity has been found in various Pd–Te compounds with distinct *T*_c_, with the highest known to date as that of PdTe (*T*_c_ ∼ 4.5 K),^47–49^ and all other known phases exhibit a much lower *T*_c_. Our observation of a slighter lower *T*_c_ (3.8 K) and a much higher *B*_c,⊥_ compared to PdTe^47–49^ is consistent with the structural characterization of it being a PdTe-type crystal but with a much lower Te concentration. It also demonstrates that our approach can achieve significant variations of atomic contents in crystals. Superconductivity is observed over the entire crystal, as implied by data taken from another pair of contacts (Figure S6).

Our work establishes a new synthetic pathway to high-quality crystals through direct on-chip chemical processes under nanoconfinement employing a novel concept of vdW nanoreactors. The two reactions demonstrated here represent only the initial examples of what vdW nanoreactors can achieve. We anticipate that many more quantum materials will soon be synthesized and characterized by using this approach. A vast array of ultrathin materials—whether exfoliated,^31–33^ deposited, chemically modified, or fabricated by other means—can be stacked to form vdW nanoreactors. Within these structures, reactions can be initiated not only by heating but also through alternative stimuli, such as high-pressure or laser irradiation, opening an expansive space for exploration.

One particularly promising direction is the search for improved superconductors and topological quantum materials that are challenging to obtain via conventional bulk synthesis. There are at least two key advantages of using vdW nanoreactors in the context of superconductivity. First, the resulting materials are microscale single crystals compatible with nanofabrication, naturally minimizing issues such as fractional superconducting volume often encountered in bulk samples in the search for new superconductors. Second, chemical reactivity under nanoconfined conditions differs significantly from that in open environments, offering access to materials with composition and stoichiometry that may be difficult to achieve using traditional methods but important to optimizing superconductivity. Another exciting avenue is leveraging vdW nanoreactors to deepen our understanding of nanoscale chemistry and molecular dynamics under confinement—a topic of broad relevance to fundamental research at small scales. By carefully selecting materials and reaction conditions, these systems can serve as well-defined platforms for studying chemical processes and material transformations in the nanoscale. The resulting insights could inform diverse fields, including biological systems, nanofluid channels, atomic-scale manufacturing, and emerging small-scale technologies.

## Supplementary Material



## Notes

Additional experimental details, methods, materials, and measurements, including figures of additional experimental data (PDF)
