# Supplementary material for: van der Waals Nanochemical Reactors
Source: Nano Lett. 2026 Jan 26;26(5):1920–6. doi: 10.1021/acs.nanolett.5c06176 (PMC12904075; doi:10.1021/acs.nanolett.5c06176)
Supplement: Supplementary file 1 [file nl5c06176_si_001.pdf]

Supporting Information

van der Waals Nanochemical Reactors

Zhaoyi Joy Zheng[1,2,‡], Haosen Guan[1,‡], Danrui Ni[3], Guangming Cheng[4], Yanyu Jia[1], Ipsita Das[1], Yue Tang[1], Ayelet J. Uzan-Narovlansky[1], Lihan Shi[1,2], Kenji Watanabe[5], Takashi Taniguchi[6], Nan Yao[4], Robert J Cava[3], Sanfeng Wu[1]*

[1] Department of Physics, Princeton University, Princeton, New Jersey 08544, USA

[2] Department of Electrical and Computer Engineering, Princeton University, Princeton, New Jersey 08544, USA

[3] Department of Chemistry, Princeton University, Princeton, New Jersey 08544, USA

[4] Princeton Materials Institute, Princeton University, Princeton, New Jersey 08544, USA

[5] Research Center for Electronic and Optical Materials, National Institute for Materials Science, 1-1 Namiki, Tsukuba 305-0044, Japan

[6] Research Center for Materials Nanoarchitectonics, National Institute for Materials Science, 1-1 Namiki, Tsukuba 305-0044, Japan

‡These authors contributed equally to this work

[*] Email: sanfengw@princeton.edu

Methods

Fabrication of vdW nanoreactors for Te growth

To prepare the vdW nanoreactors, $MoTe_2$ flakes and hBN were mechanically exfoliated on SiO_2/Si substrates. We chose 2 ~ 4 layer $MoTe_2$ which were identified under an optical microscope located in an Ar-filled glovebox. $MoTe_2$ flakes were then placed on top of a 10-15 nm thick hBN flake to form a $MoTe_2$/hBN stack on a SiO_2/Si substrate using standard 2D dry transfer techniques. The stack was subsequently placed in a furnace and treated at 280 ºC for 1 hour with O_2 gas flow at a rate of 0.1 L/min. After the $MoTe_2$ oxidization process, another hBN flake (15-20 nm thick) and a fresh $MoTe_2$ (2 ~3 layer) flake was transferred onto this Mo-Te-O oxide/hBN bottom stack. To trigger the reaction and induce Te growth, the final stack (hBN/$MoTe_2$/Mo-Te-O oxide/hBN) was placed in the furnace and heated up to 350 ºC for 30 minutes, with oxygen at a flow rate of 0.1 L/min. Growth T1-3 all followed the above process.

Fabrication of vdW nanoreactors for Pd-Te crystal growth

To prepare the vdW nanoreactors for Pd-Te crystal growth, the bottom oxidized $MoTe_2$/hBN stack was prepared using the same method as described in the Te growth. To prepare the Pd_7MoTe_2 reactant, we stack another hBN flake and a bilayer $MoTe_2$ together and subsequently placed them on a pre-deposited thin Pd source on a SiO_2/Si substrate. The Pd source was prepared using standard electron beam lithography, followed by cold development, reactive ion etching, and metal deposition to deposit the patterned Pd sources of about 20 nm thickness on SiO_2/Si wafers. This hBN/$MoTe_2$/Pd stack was annealed in a furnace at 300 °C for 1 hour under a forming gas flow (90% N_2 + 10% H_2) at 0.1 L/min to induce 2D Pd diffusion, resulting in the formation of a Pd_7MoTe_2 thin film.[42-44] The resulting hBN/Pd_7MoTe_2 stack was then picked up and transferred

onto the previously prepared oxidized $MoTe_2$/hBN stack, yielding the final vdW reactor structure: hBN/Pd_7MoTe_2/Mo-Te-O oxide/hBN. This final stack was subjected to crystal growth conditions under high temperature treatment up to 400 °C. For Growth PT1, the stack underwent two growth stages: an initial treatment at 350 °C in oxygen (0.1 L/min) for 30 minutes, followed by a second stage at 400 °C in oxygen (0.1 L/min) for another 30 minutes. Growths PT2 and PT3 both involved a first stage at 300 °C in oxygen (0.1 L/min) for 1 hour, followed by a second stage at 400 °C in forming gas (0.1 L/min) for 1 hour. Growth PT4 followed a similar two-step procedure, with the first stage at 300 °C in oxygen (0.1 L/min) for 45 minutes, and the second stage at 400 °C in forming gas (0.1 L/min) for 1 hour.

Plan-view TEM Sample Preparation

The plan-view TEM samples were prepared by transferring hBN-encapsulated (from both top and bottom) samples after final growth to TEM grids with holey silicon nitride, using the standard 2D dry transfer techniques employing polycarbonate film. The polycarbonate film left on the stack after the transfer was dissolved in chloroform for 30 min. A cartoon illustration of the sample preparation process can be found in **Extended Data Fig. 3a**.

Cross-section TEM Sample Preparation

The cross-section TEM sample were prepared by focused-ion-beam (FIB) cutting the $PdTe_{1-x}$ device (see below on the transport device fabrication) using Helios NanoLab G3 UC dual-beam FIB-SEM. A protective Carbon layer was first deposited using a gas injection system to prevent beam-induced damage during milling. The region of interest was then milled using Ga^+ ion beam and extracted using the in-situ lift-out method. The lamella was then mounted onto a TEM grid for imaging. Final thinning and polishing were performed using 2kV Ga^+ ion beam till the sample is

electron-transparent with a thickness less than 100 nm. A cartoon illustration of the sample preparation process can be found in **Extended Data Fig. 3b**.

SEM

The SEM experiments were performed using Verios 460 XHR SEM. The samples were mounted on conductive carbon tape and imaged without additional coating. SEM images were acquired under high vacuum using a secondary electron detector with an accelerating voltage of 5 kV, a beam current of 25 pA, and a working distance of 4 mm.

S/TEM and EDX

Atomic-resolution HAADF imaging and EDX mapping were performed using Titan Cubed Themis 300 double Cs-corrected S/TEM equipped with an extreme field emission gun source and a super-X EDS system. The system was operated at 300kV.

Transport Device Fabrication

To prepare transport devices for Te nanowires, the hBN encapsulated final stacks after growth were picked up and transferred onto SiO_2/Si wafers with prepatterned metal alignment markers. We used electron beam lithography to pattern metal electrodes, followed by cold development. Then we used reactive ion etching to etch through top hBN and used e-beam metal deposition to deposit metal contacts (~5 nm Ti / ~120 nm Au). For the Pd-Te device, the hBN encapsulated final stacks after growth were picked up and transferred onto SiO_2/Si wafers with prepatterned metal alignment markers. The top hBN was etched through using reactive ion etching to expose the as-grown crystals. We then employed standard electron beam lithography, followed by cold

development, reactive ion etching, and metal deposition to pattern and deposit metal contacts (~15 nm Ti / ~135 nm Au).

Transport Measurements

The electrical transport measurements for Te nanowires were performed in a Quantum Design Dynacool system with a variable temperature down to 1.8 K. Both typical four-probe and two-probe measurements showed consistent semiconducting nature of the Te crystal at room temperature. However, the large resistance due to the semiconducting gap prevents it from using the four-probe measurements at lower temperatures. We recorded reliable two-probe resistances down to lower temperatures (~ 190 K) using the standard ac lock-in technique with a low frequency (~5 Hz) voltage excitation (~ 10 mV) applied to the source electrode while recording the current at the drain while all other electrodes are floated. The electrical transport measurements for Pd-Te compound device were conducted in a dilution refrigerator with a superconducting magnet up to 8 T and a base temperature of ~ 30 mK. The resistance measurements for Pd-Te compounds were performed using the standard ac lock-in technique with a low frequency around 21 Hz and an ac current excitation of around 1 uA.

A dc current was applied to the source electrode for critical current measurements.

Supplementary Figures

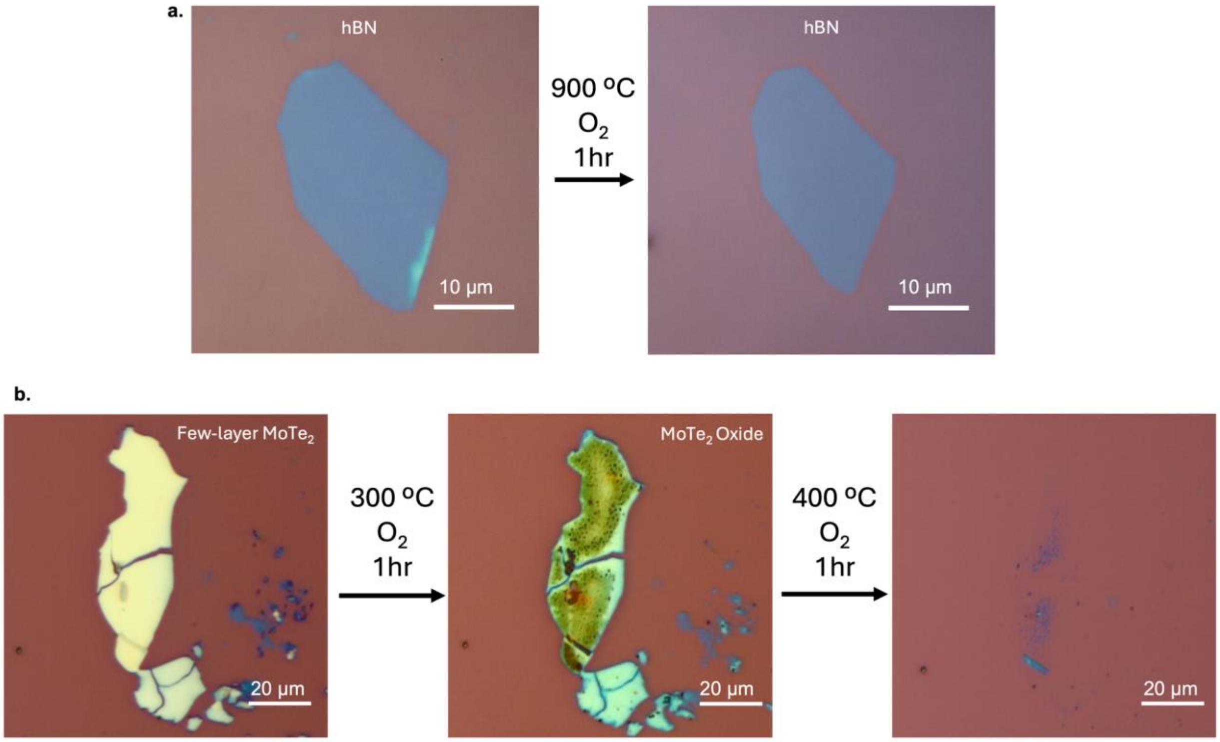

Figure S1|The impact of temperature and oxidization on hBN and $MoTe_2$ a., Optical images of a hBN flake (15 nm thick) before and after 900 ºC heat treatment in the furnace. The hBN remains stable under this high temperature, showing no evidence of oxidization. Residual glue-like contaminants at the edge are removed during the annealing process. **b.,** Optical images of exposed few-layer $MoTe_2$ (left) on SiO_2/Si substrate, after two successive heat treatments in an oxygen environment (middle & right). Without top hBN encapsulation, the oxidized $MoTe_2$ sublimed (right). The control experiments confirm the necessity of top encapsulation using inert materials--such as hBN—for maintaining material stability in the vdW nanoreactors demonstrated in our work.

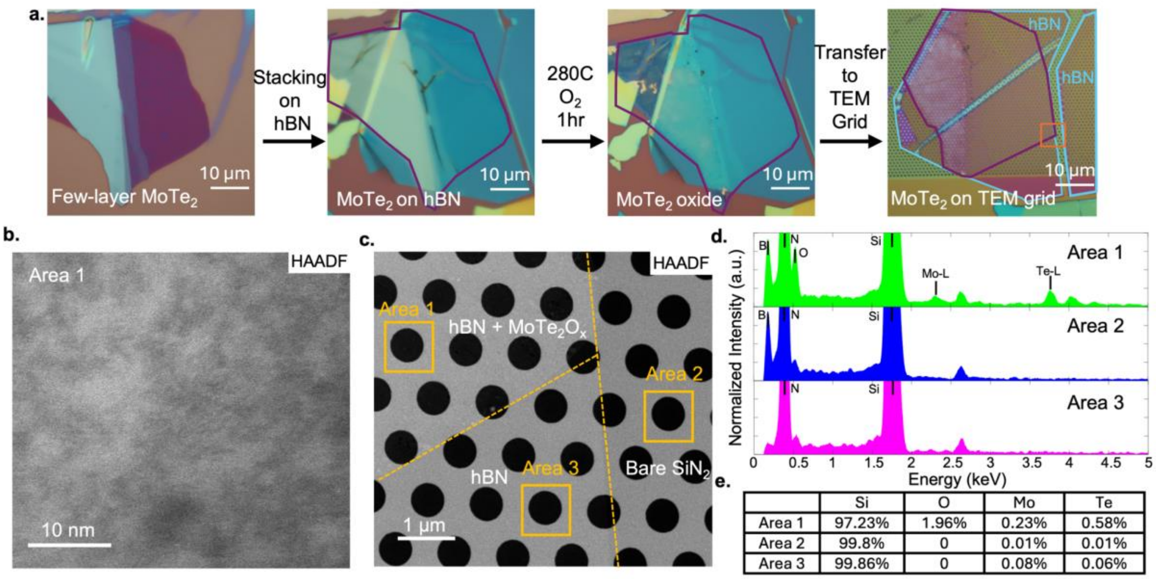

Figure S2|Charaterization of Mo-Te-O Oxide. a., Optical images at each stage of the oxidization process of a few-layer $MoTe_2$ flake and the TEM sample preparation. Purple polygons in the last three images outline approximately the shape of $MoTe_2$ or its oxides in each case. **b.,** A typical HAADF TEM image of the oxide, taken at Area 1 shown in **c.** No obvious crystalline order is observed, indicating the formation of an amorphous structure after the oxidization treatment to the original $MoTe_2$ crystal. **c.,** An HAADF image of top view of $MoTe_2$ oxide sample, with corresponding locations indicated by the orange square in the last image in **a**. Area 1 consists of $MoTe_2$ oxide on hBN, Area 2 is bare SiN_2 substrate, while Area 3 is only hBN on the substrate. The dashed line indicates the phase separation. **d.,** EDX spectra of Area 1, Area 2 and Area 3 of the spots shown in **c**, respectively. The characteristic X-ray peaks corresponding to O, Mo and Te are well resolved in Area 1, while the peak corresponding to O is absent in Area 2 and 3. **e.,** Extracted atomic fractions of Si, O, Mo, and Te for Areas 1-3, respectively. Oxidized $MoTe_2$ is roughly of the composition Mo:Te:O ~ 1:2.5:8.5, consistent with the expectation of the formation of MoO_3 and TeO_x.

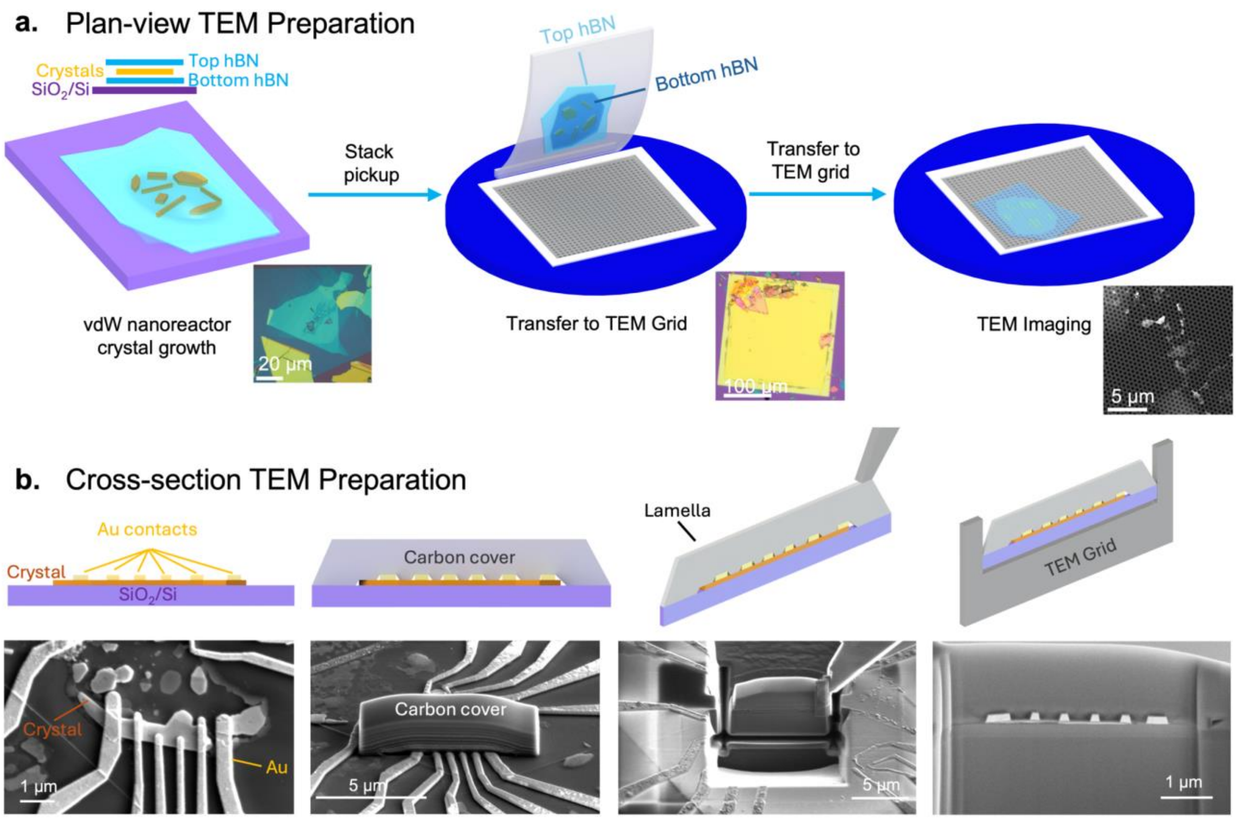

Figure S3|TEM Samples. a., Cartoon illustrations of plan-view TEM sample preparation process. The vdW nanoreator after final crystal growth (encapsulated by top and bottom hBN) is transferred onto holey silicon nitrate TEM grid. Insets are optical (the first two) and STEM (the last) images of a typical sample at corresponding stages. **b.,** Cartoon illustrations (top panel) and SEM images (bottom panel) of cross-section TEM sample preparation process. The transport device was first covered by a layer of carbon and a lamella was prepared using standard FIB milling process. The final lamella was transferred to a TEM grid.

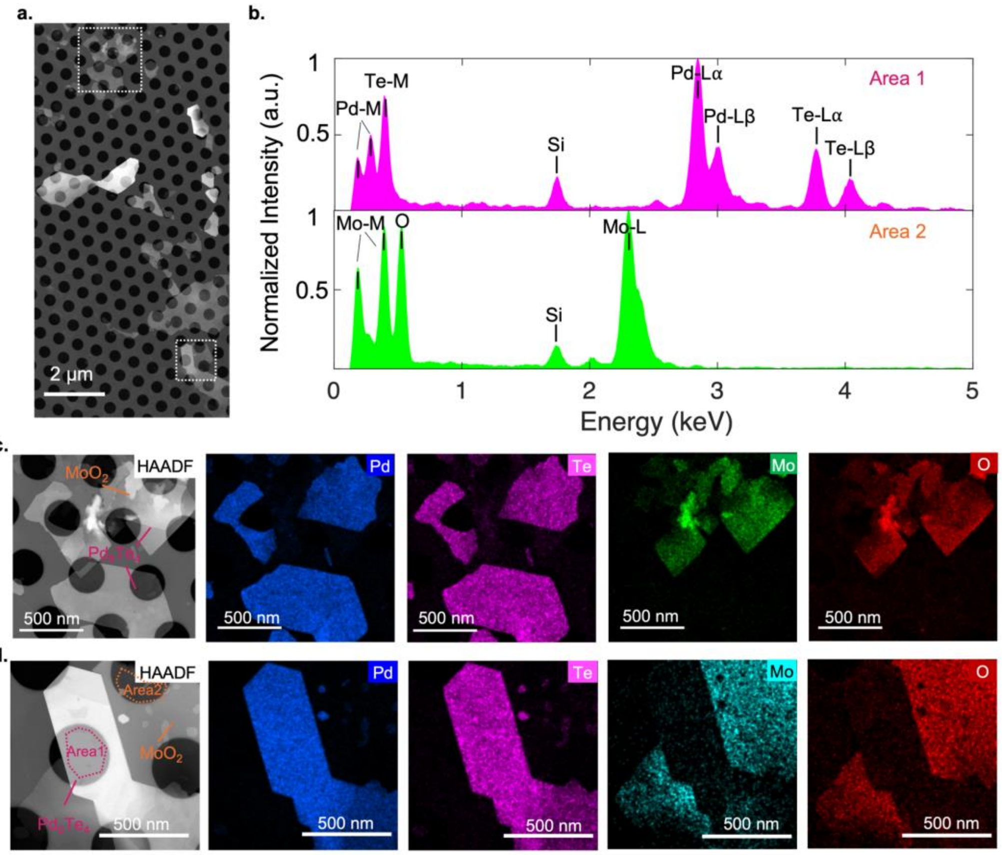

Figure S4|Additional EDX Data around two Pd_9Te_4 crystals. a., A plan-view HAADF TEM image of the sample. **b.,** EDX spectra of Area 1 and Area 2 in the sample regions shown in **d**, which zooms into the bottom white square in **a**. The characteristic X-ray energy peaks corresponding to electron relaxation to the L/M shell of Pd and Te, namely, Pd-M, Te-M, Pd-L and Te-L, are well resolved Area 1, while Mo-L and O peaks emerge only in Area 2. **c & d.,** EDX elemental mapping of Pd, Te, Mo, and O corresponding to the samples in two boxed areas (by white dashed lines) in **a**, respectively. The corresponding Pd-Te compounds are closely approximate to Pd_9Te_4, formed together with the nearby thin-film compound that is approximately MoO_2.

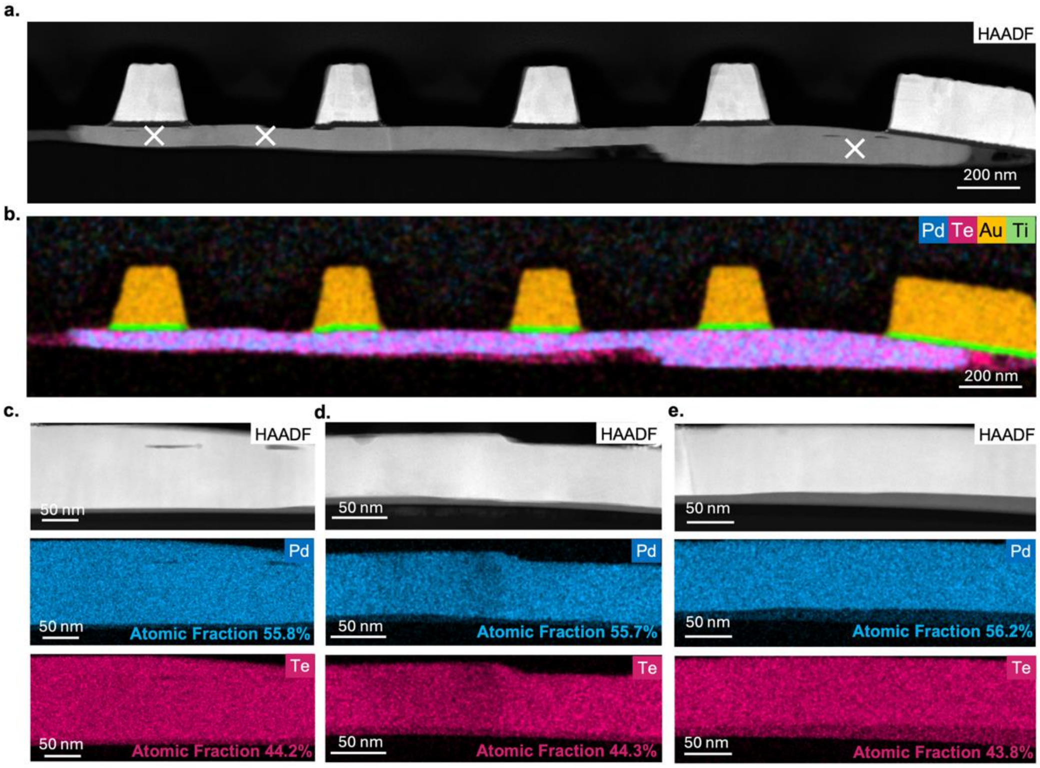

Figure S5|Additional EDX Data on $PdTe_{1-x}$ a., HAADF TEM image of cross-section view on $PdTe_{1-x}$ with deposited Au electrodes. **b.,** EDX elemental mapping of all elements detected, including Au and Ti from electrode deposition and Pd and Te from the as-grown crystal. **c-e.,** Cross-sectional EDX analysis of the slice at three different locations as indicated by the white cross in **a**, highlighting the mapping of Pd and Te for the crystal. The observed atomic ratio is indicated in each mapping, suggesting a high uniformity across the device.

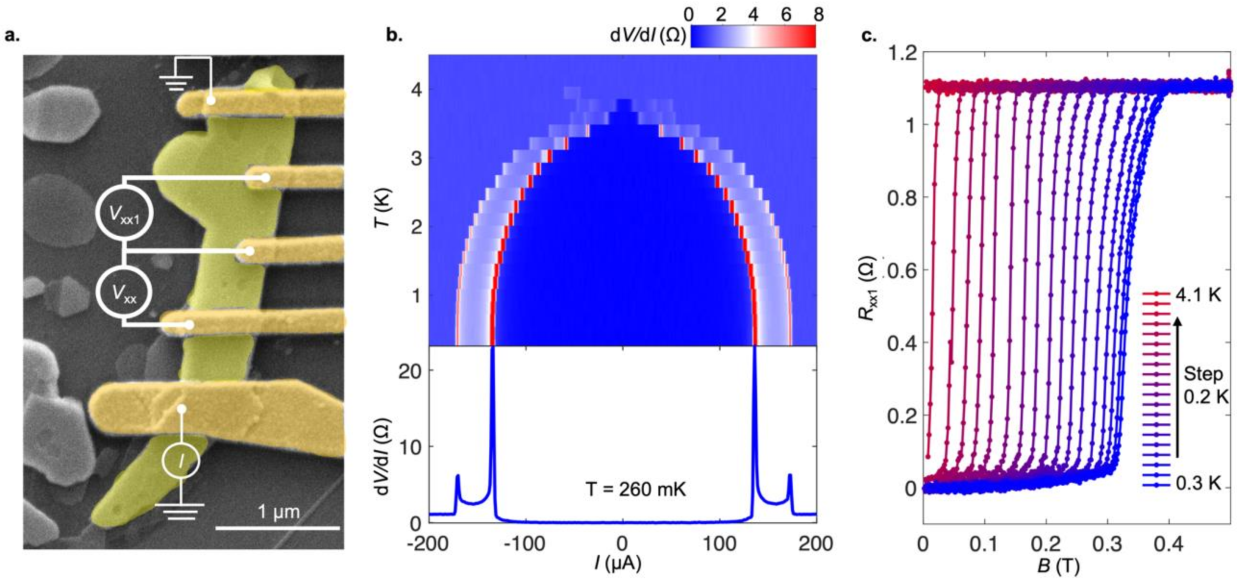

Figure S6|Additional Transport Measurement for $PdTe_{1-x}$ a., A false-colored SEM image of a device made of as-grown $PdTe_{1-x}$ with deposited Au electrodes. **b.,** Differential resistance d*V*/d*I* as a function of applied DC current (*I*), taken at different *T*, showing the *T*-dependent critical currents measured from Vxx_1, as illustrated in **a**. The bottom panel is a single curve d*V*/d*I* *vs* *I* taken at *T* = 260 mK. **c.,** R_{xx1} as a function of $B_{\perp}$, taken at various *T* as indicated by the color.
